# A Case Report of Clozapine-induced Symmetrical Drug-Related Intertriginous and Flexural Exanthema with cross-reactivity between Clozapine and Quetiapine

**DOI:** 10.1192/j.eurpsy.2023.2310

**Published:** 2023-07-19

**Authors:** S. C. Lim, A. Lijo

**Affiliations:** Psychiatry, Institute of Mental Health, Singapore

## Abstract

**Introduction:**

Symmetrical Drug-Related Intertriginous and Flexural Exanthema (SDRIFE) is characterised by a distinctive pattern of erythematous and symmetrical rash over the gluteal and intertriginous regions after exposure to certain systemic medications. This is an uncommon condition which has been thought to be due to a Type IV delayed hypersensitivity reaction. Literature on clozapine-induced SDRIFE remains scarce, and reports of cross-reactivity among anti-psychotics are limited as well.

**Objectives:**

To present a clinical case of Clozapine-induced SDRIFE with cross-reactivity between Clozapine and Quetiapine.

**Methods:**

We describe a case of a lady with Treatment Resistant Schizophrenia who developed erythematous lesions with desquamation over her skin fold regions and buttocks within two months of Clozapine initiation.

**Results:**

In our case, the lady was diagnosed with SDRIFE secondary to Clozapine. Clozapine was ceased, and the rashes resolved completely within a week. However, her psychiatric condition continued to worsened and she was trialed on Quetiapine. Unfortunately, she developed angioedema of the lips which necessitated a cessation of Quetiapine.

**Conclusions:**

This case report illustrates the importance of recognising this rare condition, which can be readily treated by withdrawal of the culprit drug. Given that Clozapine and Quetiapine are structurally similar and fall under the class of Dibenzodiazepines, physicians should also be aware of the possibility of cross-reactivity among anti-psychotic medications leading to hypersensitive reactions.

**Disclosure of Interest:**

None Declared

